# Social‐Therapeutic Custodial Treatment of Individuals Who Committed Sexual Offences: A Comprehensive Controlled and Multicentred Evaluation

**DOI:** 10.1002/cbm.70029

**Published:** 2026-04-01

**Authors:** Friedrich Lösel, Eva Link, Lena C. Carl

**Affiliations:** ^1^ Institute of Psychology Friedrich‐Alexander‐Universität Erlangen‐Nürnberg Erlangen Germany; ^2^ Institute of Criminology University of Cambridge Cambridge UK

## Abstract

**Background:**

In‐prison treatment of persons who committed sexual offences often showed nonsignificant, small or sometimes even negative effects, particularly in sexual recidivism. Various reasons for this situation seem to be relatively short and standardised group‐based programmes, isolated implementations and insufficient attention to context factors and needs for replication.

**Aims:**

This study contains a controlled evaluation of males who had committed serious sexual offences and received treatment in German social‐therapeutic prison units (STUs) versus a control group from regular prisons without treatment. STUs offer a broad range of treatment (including CBT) and rehabilitation measures during about 2 years of incarceration.

**Methods:**

Valid recidivism data were available for 1245 individuals, of which 710 were treated (TG) and 535 were not treated (CG). In addition to the individuals from STUs, there was also a subsample that received individual or group treatment in normal prisons. The mean follow‐up period after release was 9.3 years. As there were expectable differences between treated and untreated persons, a comprehensive propensity score matching (PSM) of relevant variables was applied that led to balanced equivalence of TGs and CGs. In addition to various criteria of recidivism (e.g., general, sexual, violent), we developed a harm index of seriousness. Offender characteristics were assessed by the regular documentation form of the Criminological Service in Bavaria that also contained the Static‐99 risk measure. For the assessment of therapeutic context factors, prisoners and staff filled in the EssenCES on the institutional climate in the STUs.

**Results:**

There were significant treatment effects on general recidivism (TG = 35.9% vs. CG = 41.4%) and nonsignificant tendencies on sexual recidivism (6.0% vs. 8.0%) and other outcomes. In contrast to general recidivism, sexual recidivism did not occur later after release. There were more promising results in a more recent cohort than in a previous cohort, and the sexual harm of treated persons decreased post‐treatment (as compared to pretreatment). Men who had committed rape of adult victims showed more violent and less sexual recidivism than individuals who had victimised children. The seven STUs differed substantially in recidivism and dropout rates. These differences were related to the risk level of the inmates, but prison climate was also associated with outcome differences. On average, there were both similarities and differences in the climate ratings by inmates and staff. Most individuals from the STUs got a legal order for relapse prevention in community treatment centres after release. However, this did not lead to a booster effect, whereas persons who were not treated in custody benefitted from therapeutic aftercare.

**Conclusions:**

The results of a comprehensive social‐therapeutic treatment of prison inmates who had been sentenced for sexual offences showed multifaceted patterns of results. Offender variables played an important role in the outcomes. Individuals who had victimised children were particularly assigned to STUs, and persons with a not generally assessed paraphilic disorder may be relevant for less promising results in sexual recidivism. There were also hints that the intensive treatment in STUs may not be superior to therapeutic interventions in regular prisons. Based on our findings, differentiated selections of groups, treatment contexts and outcome measures are suggested for further development of successful treatment strategies in custody.

## 1 Introduction

In many countries, sexual offending is a very serious but controversial issue. On the one hand, governments and legal systems aim for tough punishment and secure custody to protect the population. On the other hand, they follow a second pathway and aim to reduce risks of (sexual) reoffending by measures of correctional treatment. For individuals who carried out serious sexual offences, both approaches are connected when treatment is implemented in prisons. There are numerous discussions on correctional treatment that refer to penal philosophy, labelling theory, economy, public opinion etc. (Lösel 2012). Beyond these more or less theoretical discussions, a key question is the effectiveness of custodial treatment of individuals who sexually offended.

Various systematic reviews and meta‐analyses (MAs) have addressed this question. Schmucker and Lösel (2015, 2017) integrated 28 eligible studies with a treated group (TG, mainly cognitive‐behavioural treatment, CBT) and an untreated control group (CG). Only studies at Levels 3–5 on the Maryland Scientific Methods Scale (SMS) were eligible; that is, there was probably equivalence of TG and CG. The effects on reoffending in the primary studies varied substantially, but the average was significantly positive. The mean reoffending rates were 10.1% (TG) versus 13.7% (CG) for sexual offences and 32.6% versus 41.2% for all offences. Studies with smaller samples, groups at higher risk, better descriptive validity, more individualised programmes (instead of a pure group format) and treatment in the community showed a larger mean effect size (ES). The mean effect size (ES) for treatment in custody was nonsignificant.

Holper et al. (2024) published an update of the MA of Schmucker and Lösel (2017). They did not carry out a new search but added several more recent studies. Their MA contained 37 primary studies. Most results were consistent with our study, and there was also similarity in various moderators. Treatment specialisation showed a differential effect, and the difference between custody and community was somewhat less distinct.

In a broader MA of treatment for various types of offenders, Gannon et al. (2019) also evaluated specialised treatment of individuals who committed sexual offences. They included 44 primary studies, partially at lower design quality (e.g., without matching). The mean sexual recidivism rates were very similar as in our MA (TG = 9.5%, CG = 14.1%). There was also a positive effect on other forms of reoffending. The effects in prisons and communities did not differ significantly. There were somewhat stronger effects when treatment integrity was higher and psychologists were involved, but these findings were based on few studies due to missing data.

In a recent ‘umbrella review’ (a meta‐evaluation of MAs), Koehler and Lösel (2025) compared meta‐analytic results of offender treatment in custody and in the community. As groups who are treated in prisons differ from those who receive treatment in the community, both contexts cannot be compared directly. Therefore, we calculated the ESs between the TGs and CGs in custody and between the TGs and CGs in the community. The results for the groups who offended sexually were most consistent: All MAs showed substantially larger ESs in the community. The mean ES in custody was small but still significant and based on more homogeneous results than in the community (where treatment contexts and conditions are more varying).

Although the mean ESs of treatment of offenders who had committed sexual offences (abbreviated SOT) in the above‐mentioned and other MAs were positive (i.e., less sexual recidivism in the TGs), the integrated primary studies had very heterogeneous results. Some methodologically well‐evaluated programmes showed significant reductions in sexual recidivism (e.g., Olver et al. 2009), whereas others revealed no significant effect (e.g., Grady et al. 2017) or even negative outcomes. A famous example of the latter is the very large evaluation of the widely implemented prison‐based *Sex Offender Treatment Programme* (SOTP) in England and Wales. The evaluation by a team of the Ministry of Justice (Mews et al. 2017) used propensity score matching (PSM) to compare a TG of 2562 convicted sex offenders with a CG of 13,219 untreated prisoners. The sexual reoffending rates were 10.0% for the TG and 8.0% for the CG. For child‐images reoffences, the respective rates were 4.4% (TG) and 2.9% (CG). The British mass media scandalised the ‘waste of £100 million for a prison programme that increased recidivism of sex offenders’, and the government immediately stopped the delivery of SOTP. Although the results of Mews et al. (2017) were untypical, they partially fitted moderators in the Campbell Collaboration MA of Schmucker and Lösel (2017): less positive (although not negative) results for treatment in prisons, very large samples, group‐only treatment format, nationwide implementation in daily practice and independent evaluators.

The mixed findings underline difficulties of proving successful SOT in prisons. In the last decades, the official recidivism rates for sexual offences became rather low (Lösel et al. 2023; Lussier et al. 2023). This is good news for societies and potential victims, but ‘floor effects’ facilitate nonsignificant results in treatment evaluations. In addition to dichotomous recidivism rates, more sensitive criteria such as seriousness or time for reoffending should be considered. We must also be aware of basic therapeutic constraints of treatment in prisons: Reality tests in risk situations and transfers of learning are restricted because there are no children or females except prison staff.

Most current SOT programmes apply CBT concepts and adhere to the overarching risk‐need‐responsivity (RNR) model. The RNR model and ‘what works’ literature on correctional treatment have sometimes been criticised, for example, by proponents of the Good Lives Model (GLM; Ward and Stewart 2003). Most recently, an ‘umbrella review’, that is, an integration of meta‐analyses on offender treatment, suggested that RNR should not be used any more (Fazel et al. 2024). However, a detailed analysis of this study showed serious weaknesses that did not at all justify its far‐reaching conclusions (McGuire et al. 2025). Although the Fazel et al.'s (2024) article did not specifically address the field of SOT, it was also received by practitioners in this field who are experimenting with different approaches. However, from an empirical point of view, concepts such as the GLM do not yet have a similar evidence base as RNR (Netto et al. 2014; Zeccola et al. 2021). In practice, strength‐oriented aspects of GLM are already included in the RNR model (e.g., Andrews et al. 2011), and its fully justified emphasis on strengths and therapeutic alliances are considered in current CBT/RNR‐oriented approaches (e.g., Abracen et al. 2011; Olver et al. 2020).^1^ They also include knowledge on desistance (Farrall and Calverley 2005; Maruna 2001) and resilience (Lösel and Bender 2003; Lösel and Farrington 2012).

The above‐mentioned and other concepts of offender treatment should not be seen as polarised alternatives or paradigms but as stimulation for both broader and more differentiated approaches (probably with RNR and CBT evidence‐based backbones). As a consequence of nonsignificant or very small treatment effects of many programmes in custody, authors suggested widening the perspective of isolated programmes and ‘silo’ approaches to the complex concepts of offender rehabilitation (Maguire et al. 2010). These concepts emphasise the many aspects of rehabilitative influences beyond single CBT programmes etcetera. In a stepwise process, they adequately consider the numerous features of the programme, context, offender and evaluation method that have an impact on effective correctional treatment (Lösel 2018). A typical example is the research on a humane prison climate that showed not only positive effects on prison violence but also (on the aggregate level) on recidivism (Auty and Liebling 2020, 2024).

### 1.1 Social‐Therapeutic Prisons

The present study follows this perspective and evaluates the complex treatment in German social‐therapeutic prisons for the group of individuals who had been sentenced for sexual offences. These prisons are partially similar to prison‐based therapeutic communities that are internationally mainly used for drug‐addicted or personality‐disordered offenders. There are different models of therapeutic communities (Cullen and Woodward 1997). Typically, clients have close informal relationships, share information, are committed to learning, strive for open problem resolutions, are sensitive to the psychodynamics of individual and group processes and follow boundaries concerning roles, time and place (Kennard 1998). A prominent example is Grendon prison in England (Genders and Player 1995, 2010).

German social‐therapeutic prisons are partially similar as well as different to Anglo‐American hierarchical therapeutic communities in prisons. They share basic characteristics with regular (‘normal’) prisons in Germany. Originally, they were mainly designed for personality disordered, serious and violent repeat offenders, but their development stagnated due to costs, the ‘nothing works’ discussion, labelling and other reasons (Lösel et al. 1987). Because of a criminal law reform in 1998, there was a ‘revival’, and new social‐therapeutic prisons were established with a particular focus on sexual offenders. Individuals who received a prison sentence of more than 2 years for a sexual or other violent offence ought to be transferred to social‐therapeutic facilities. There are now 71 social‐therapeutic institutions in Germany, some specifically for young or female offenders. They are mostly organised as widely independent social‐therapeutic units (STUs) attached to a larger regular prison. Eligible inmates of regular prisons can apply for transfer to an STU, and after an assessment process and a short probation period, they typically stay there for about 2 years.

STUs have a similar security regime as regular prisons but employ more staff per inmate and, in particular, more social service personnel such as psychologists, psychotherapists, social workers and (rarely) physicians. The inmates have to work, but time is allocated for group and individual psychotherapeutic sessions, social training free contact among inmates, basic and further education, sports and other leisure activities etc. The therapeutic processes vary somewhat between different STUs with regard to treatment contents and dosage. Typically, STUs follow a CBT/RNR approach that contains structured and more individualised therapy and training sessions (e.g., on anger management and self‐control). In some units, additional elements of systemic, insight‐oriented, psychodynamic, schema, music or arts therapy are offered. Contact with family members and support from local volunteer organisations is promoted. In a stepwise process, inmates can progress into a more open form of custody; for example, they can get a leave of absence, work in a business outside, and buy and prepare their own food. There is a more intensive preparation for release and resettlement than in regular prisons and often inmates get released after two thirds of their sentence. In cases of serious misconduct or on their own request, STU prisoners can be retransferred to regular prisons.

If not assigned to an STU, for example, due to deficient treatment motivation or limited language skills, individuals convicted of sexual offences may instead participate in specialised individual or group‐based treatment within the regular prison system, if resources are available. Individual treatment involves consultations with psychologists or external psychotherapists on a weekly or biweekly basis and is individually tailored to the needs of each client. Group‐based treatment involves participating in a German version of SOTP.

### 1.2 Research Questions

In the following, we present a methodologically controlled, multicentred evaluation of treatment in STUs for individuals who were sentenced for sexual offences. As often requested in the literature, we are not investigating a demonstration project but daily routine treatment in practice. According to the above introduction, our main research questions were the following:

1. Is treatment in STUs effective in reducing sexual and other forms of reoffending?
2. Do different outcome criteria show different results?
3. Are there outcome differences over time that may indicate treatment progress?
4. Are individual characteristics of the inmates relevant for different outcomes?
5. Are potential effects homogeneous and generalisable across different institutions?
6. Are institutional climate characteristics relevant for effectiveness?
7. Can postrelease ambulatory treatment increase custodial treatment effects?

As in most evaluations of treatment for individuals with serious sexual offences, we could not apply a randomised design. Therefore, we apply rather valid methods of propensity score matching (PSM) for balancing equivalence between TGs and CGs. In addition to dichotomous official reoffending rates, we investigate criteria of seriousness and time of reoffending as well as differences between treatment cohorts and offender types. As our main focus is on recidivism after treatment in STUs, other findings can only be reported briefly. This applies to data on correctional treatment in regular prisons that we received as a ‘bycatch’ from the Federal German Registry and the documentation form of the Criminological Service of the Bavarian correctional system (Ministry of Justice).

## 2 Method

### 2.1 Participants

Our project extended and intensified a smaller previous study of the Criminological Service of the Bavarian Justice Ministry. We included all males who received a prison sentence of more than 2 years for a sexual offence and were released between 2004 and 2015. As described above, treatment in an STU is legally mandatory in these cases. Therefore, in our research plan, we did not expect many equivalent individuals for a control group (CG) in comparison to the treated group (TG). However, as there is often a discrepancy between law in the books and law in practice, we surprisingly got data on many individuals who had fulfilled the legal treatment criteria but were not transferred to an STU from the regular prisons where they were originally placed for legal reasons. Our detailed analysis of these cases showed various reasons for not being transferred to an STU, for example, not enough vacant places in STUs, organisational problems such as professional training in a regular prison, individual rejection by specific STUs, distance of an STU from the family home or other reasons why inmates refused a treatment offer. According to this background of the untreated individuals, we expected and found that the TG and CG were originally not fully equivalent (as in an RCT). We, therefore, applied matching techniques (PSM, see below).

For information on recidivism, we got basic data for 1787 individuals from the German Federal Central Register. Because of various reasons (e.g., emigration, lower sentence, death, doubled cases), we finally received valid recidivism data on *n* = 1245 individuals. Of these, 710 had been treated and 535 were not treated. Of the treated individuals, 449 participated in the comprehensive STU programme, whereas 261 got some individual (96) or group therapy (165) in regular prisons (which was not the legally required treatment in an STU). The average duration of treatment in STUs was *M* = 27.5 months (SD = 11.9). The intervention in regular prisons lasted *M* = 11.8 months (SD = 8.1) for individual treatment and *M* = 18.7 months (SD = 12.3) for group programmes. Overall, the mean age of treated versus untreated individuals was *M* = 43.2 years (SD = 11.6) versus *M* = 44.9 years (SD = 12.2) and was similar for all treated subgroups. However, the three subgroups differed in their risk level: The Static‐99 mean scores were *M* = 2.01 (SD = 0.99) for treated persons in STUs, *M* = 2.38 (SD = 0.95) for individual therapy and *M* = 1.6 (SD = 0.80) for group therapy in regular prisons (*F* (2,351) = 10.27, *p* < 0.001). Our project extended and intensified a smaller previous study of the Criminological Service of the Bavarian Justice Ministry. We gathered information on all sexual offenders who served a prison sentence of more than 2 years in Bavaria and were released between 2004 and 2015.

Overall, there were differences between treated and untreated groups, that is, for lack of occupational qualification (TG = 31.45, CG = 43.6%), migration background (TG = 17.9%, CG = 37.4%), serious alcohol abuse (TG = 24.8%, CG = 31.8%), being a victim of abuse in one’s own childhood (TG = 18.4%, CG = 7.9%), sexual deviance/paraphilia (TG = 15.1%, CG = 6.0%; with many missing data), previous incarceration (TG = 17.8%, CG = 28.6%), mean age at first sexual offence (TG = 34.2, CG = 35.6 years), physical harm to victims (TG = 17.6%, CG = 26.1%) or male victims (TG = 18.1%, CG = 13.1%). Individuals with only child victims were more frequently treated (64.3%) than those with only adult victims (47.7%). Because of such differences in biographical and psychological variables, we applied a propensity score matching (PSM) procedure for the analyses of recidivism (see next paragraph). Thus, we controlled for selection effects and could assume equivalence between the final TGs and CGs. We also used a conservative intent‐to‐treat analysis that counted all individuals who dropped out after the probationary period in the TG. The average follow‐up period after release was *M* = 9.3 years (SD = 3.5 years).

### 2.2 Propensity Score Matching

According to Rosenbaum and Rubin (1983), PSM enables an approximation to randomisation in quasi‐experimental designs. There are various statistical techniques for matching treated and untreated groups, for example, stratification based on risk levels, direct matching (perhaps with callipers of equivalence in confounding variables) or control by multiple regression analyses. We applied a version of PSM that aims to come as close as possible to random assignment, as it controls for a large number of confounding variables. Like an RCT, it clearly separates the design from the analysis of the outcome and enables an evaluation of the degree of equivalence (balance) between TG and CG (Kuss et al. 2016). In a ‘true’ random allocation (in a large sample), the probability of an individual being assigned to the TG or the CG is *p* = 0.50. This leads to an equivalent propensity score (PS), whereas in quasi‐experiments it must be achieved statistically. There are different methods of balancing the PSs between TG and CG (Austin 2011). We used the method of *inverse probability of treatment weighting* (IPTW, Austin and Stuart 2015). Hereby, each individual in the TG gets a weight of 1/PS and each one in the CG of 1/(1‐PS). To keep the sample sizes constant, we calculated stabilised weights (Xu et al. 2010) for our recidivism analyses. Although some experts on PSM recommended including as many variables as possible to simulate randomisation, we considered some critical arguments (e.g., King and Nielsen 2019) and focused on matching variables that are relevant for our topic of treatment and recidivism. We included 36 variables from the regular data entry form for sexual offenders in Bavarian prisons. This assessment system contains characteristics of the individual biography (e.g., age, marriage, child abuse, migration), developmental background (e.g., broken family, foster family, residential care), educational and professional career (e.g., graduation at school, professional qualification, unemployment), personality features (substance abuse, mental health problems, reduced responsibility), offence history (youth crimes, incarcerations, previous sexual offences, remand sentences), details of the index sexual offences (type of offence, kind of victims, violence, weapons) and overall Static‐99 risk scores etc. As some documented variables had several categories, we partially used dummies so that the total number of matched items was 58. For reasons of space we cannot describe all PSM variables here. We will send the whole list on request. We only included variables that were assessed before imprisonment. Although the percentage of missing data was low (< 10%), some relevant variables were not sufficiently documented in the system. For these few variables, we applied the EM algorithm for imputation (Kleinke et al. 2011). The 36 (58) variables were included as predictors in a logistic regression analysis with TG versus CG as the dependent variable. The resulting PSs were different between both groups (standardised mean difference *M* = 12.78, SD = 9.94). When we applied the IPTW procedure, the weights varied between 0.44 and 7.18. Only 19 individuals had to be excluded because their PS score was outside the common‐support area (where the PSs of both groups overlapped). The standardised mean difference decreased to *M* = 1.76 (SD = 1.66), and only one value was at the common threshold of 10. Accordingly, the balance diagnosis suggested equivalence between the weighted TGs and CGs. This was similar for all recidivism analyses we will report below.

### 2.3 Instruments

#### 2.3.1 Bavarian Documentation System

For the PSM, we used the regular data entry form for individuals who committed sexual offences that had been developed by the Criminological Service of the Bavarian correctional system (Ministry of Justice). This instrument contains 72 differentiated items in five domains: (i) biographic (11 items, e.g., age, family of origin, migration, occupational qualification); (ii) criminological (10 items, e.g., juvenile delinquency, criminal record, previous incarcerations); (iii) psychological and psychiatric (11 items, e.g., criminal responsibility, personality disorder, alcohol or drug dependence, intelligence); (iv) sexual offending (19 items, e.g., child abuse or rape, type and number of victims, hands‐on/off delicts, use of weapons, denial); (v) imprisonment and treatment (13 items, e.g., previous treatments, probation, relaxations, violence in custody); and (vi) after release (8 items, e.g., family support, accommodation, conditions of probation). The questionnaires had been regularly filled in by experienced staff members (i.e., psychologists) in the prisons at the time of release. For a number of individuals and various items, there were some missing data. These uncertainties were reduced by additional reports from prison staff and inspections of prisoners' files by our research team. In spite of these intensive efforts, some information could not be procured, for example, detailed psychological and psychiatric data on the subgroup of inmates in regular prisons outside the STUs. When only a few individuals had missing items, we carried out an imputation (see above).

#### 2.3.2 Static‐99

For some of our analyses, we used the Static‐99 risk assessment instrument (Hanson and Thornton 2000) as a control variable. We applied a German translation of the original version that fitted better to German data than the slightly revised coding (Rettenberger et al. 2013).

#### 2.3.3 Basis Characteristics of Treatment in Social‐Therapeutic Prisons

We developed our own questionnaire on the structural and therapeutic characteristics of the seven STUs that were included in our study. This instrument was partially based on the regular annual surveys of the German Criminological Centre (KrimZ; Spöhr 2009). It addressed, for example, information on the institution's size, inmate selection, assessment procedures, treatment programmes, supervision, education programmes, job opportunities, leisure facilities, external contacts and aftercare. The structured questionnaire was filled in by the heads of the institutions.

#### 2.3.4 Institutional Climate in the STUs

In our survey of the institutional climate, we used the German version of the *Essen Climate Evaluation Schema* (*EssenCES*) (Schalast and Tonkin 2016). This internationally widely distributed, rather short instrument contains 15 items that are rated on a 5‐point scale (from ‘never’ to ‘completely’). The *EssenCES* could be used for both inmates and staff in the seven STUs. Overall, the return rates of the surveys were very satisfactory: 92% for the inmates and 73% for staff members (with somewhat higher rates for social service staff). The *Total EssenCES Score* has a potential range between 0 and 20. Its reliability in our study was Cronbach’s *α* = 0.83, somewhat higher for the inmates than for staff. There are three subscales: *Inmates' Mutual Cohesion and* *Support* (5 items, e.g., ‘The inmates care for each other’; *α* = 0.83), *Experienced Safety* (5 items, e.g., ‘Some inmates are so excitable that one has to be particularly careful in handling them’; *α* = 0.73) and *Support from Staff* (5 items, e.g., ‘Staff members take much time for the inmates’; *α* = 0.82).

#### 2.3.5 Recidivism

As described in the sampling section above, we used the data from the Federal Central Register on official recidivism. The data were intensively checked for completeness and validity. We included the following types of recidivism: (a) *general recidivism* (any conviction for an offence after release from prison; (b) *sexual recidivism* (any new sex offence, e.g., rape, sexual assault, sexual child abuse, indecent images); (c) *violent recidivism* (any new nonsexual violent offence, e.g., homicide, assault, robbery); and (d) *very severe recidivism* (any new conviction of at least 2 years of imprisonment, custody in a forensic clinic or preventive detention).

The latter is not only a measure of the respective offence’s seriousness but is also influenced by an individual's criminal career that is considered in the court's sentencing decision. For an additional analysis of the seriousness of (sexual) reoffending, we developed a harm index (similar to the Cambridge Crime Harm Index, Sherman et al. 2016). We used the range of sentences that are provisioned in the German Criminal Code and applied the following formula: severity score = (minimum sentence in months + maximum sentence in months)/2 (Lauchs et al. 2020). For some offences, no minimum or maximum is specified. Here, we set 1 month and 180 months as thresholds. One hundred and eighty months were also coded for life sentences that end after 15 years at the earliest.

## 3 Results

### 3.1 Treatment and Recidivism (Research Questions 1–3)

We compared the reoffending rates for TGs and CGs in the four main outcome criteria. Table 1 shows the respective results. As there were no significant outcome differences between treatment in STUs and interventions in regular prisons, we also report findings for treatment in general (χ^2^(2) varied between 0.19 and 4.44; all *p* > 0.05).

**TABLE 1:** Recidivism rates of the PSM‐matched treatment and control groups.

	*n*	General (%)	Sexual (%)	Violent (%)	Very severe (%)
TG (all)	711	35.9^a^	6.0	13.6	7.5
CG	520	41.4	8.0	14.3	9.1
TG (STU)	449	40.1	7.3	18.1^b^	10.9
CG	532	41.3	7.7	14.2	9.2
TG (regular prison)	261	34.1^c^	4.1	12.9	6.3
CG	582	40.3	5.8	13.6	8.0
TG (cohort 2004–2008)	352	39.3	7.3	12.0	7.6^d^
CG (cohort 2004–2008)	298	45.3	8.7	14.2	12.0
TG (cohort 2009–2015)	375	24.2^e^	4.1	5.7	5.5
CG (cohort 2009–2015)	277	31.7	6.0	7.0	8.2

*Note:* STU: treatment in social‐therapeutic units; regular prison: individual or group treatment; very severe recidivism: prison sentence of more than 2 years or confinement in a forensic hospital.

^a^ χ^2^(1) = 3.80, *p* = 0.05.

^b^ χ^2^(1) = 2.75, *p* = 0.10.

^c^ χ^2^(1) = 2.75, *p* = 0.10.

^d^ χ^2^(1) = 3.56, *p* = 0.06.

^e^ χ^2^(1) = 4.52, *p* < 0.05; tests two‐sided; all other comparisons nonsignificant.

**FIGURE 1:**
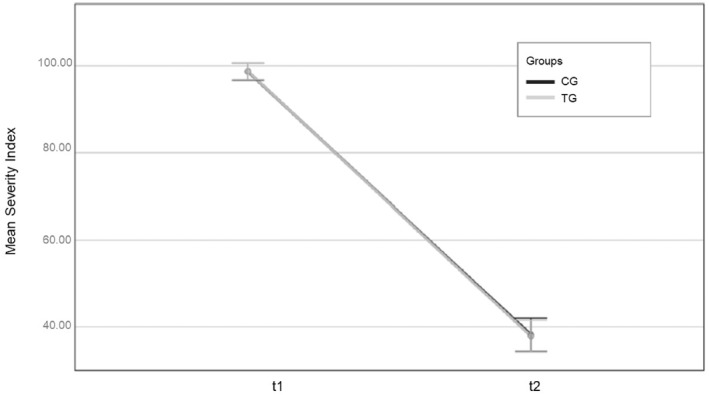
Difference in the harm level of index offences before incarceration and the first reoffence after release. The lines for the treated and untreated groups are nearly identical.

In spite of substantial numbers of participants, most of the comparisons between the matched TGs and CGs showed nonsignificant differences. The only statistical effect at *p* = 0.05 concerned general recidivism in all groups. The recidivism rates for sexual offences were particularly low, which led to a floor effect and revealed no outcome differences. Treatment in an STU was not more effective than the individual or group treatment in regular prisons. As mentioned above, these individuals were a bycatch of the German Registry on recidivism and the Bavarian prison documentation system. They were not included in our original research design and are an example of what may occur in evaluations of routine practice (instead of demonstration projects). We briefly described these groups above but did not have much detail on these groups in the Bavarian documentation system so that they were not included in our research questions. The overall harm level in reoffending decreased substantially from the index offence before incarceration to the first reoffence after release. It was nearly an identical change in the TG and CG (Figure 1) and thus only revealed a time and not a group effect. When we specifically analysed the smaller subgroup with a sexual reoffence (*n* = 48), we observed a similar tendency towards less harm in reoffending than in the prior index offence. The treated individuals showed a similar post‐treatment harm as the CG, but they decreased from a higher pretreatment level. This resulted in a significant interaction effect of time and group (*F*(1) = 3.98, *p* < 0.05; for further details see Link and Lösel 2022).

Table 1 shows that the earlier treatment cohorts (release 2004–2008) and our more recent data (release 2009–2015) revealed a consistent (although mostly nonsignificant) tendency of better treatment effects in the latter cohort. The follow‐up periods were very similar (*M* = 80.83 vs. *M* = 78.5 months), and the tendency of better recent effects remained when we set a fixed follow‐up period of 3 years for both cohorts. The earlier cohort had a slightly higher mean score in the Static‐99; *M* = 2.60 (SD = 2.06) versus *M* = 2.36 (SD = 2.19), *t* (1, 303) = 2.19, *p* < 0.05. However, after control of risk, there was still a better outcome in the recent cohort for general recidivism (Exp(*β*) = 0.59, *p* < 0.05) and violent recidivism (Exp(*β*) = 0.46, *p* < 0.05).

#### 3.1.1 Time at First Reoffending

According to the age‐crime curve, the time of reoffending could be an indicator of treatment success. Therefore, in addition to the prevalence of recidivism, we assessed the time of the first official offence after release from prison without any intermediate sentence. About one third of individuals recidivated within the first year after release and about a half within the first 2 years. The average time until any first offence was *M* = 32.5 months (SD = 31.4; *Md* = 23). The findings for sexual and violent offences were similar to those for general reoffending. Table 2 shows the means and standard deviations of the times of first recidivism for the categories where we received exact data from the central registry.

**TABLE 2:** Time until first reoffence of the individuals who recidivated in the TG or CG.

Type of reoffending	*n*	Treated *M* (SD)	Untreated *M* (SD)	*t*‐test	Effect size (d)	Significance (*p*)
General	473	36.1 (33.5)	28.6 (28.5)	−2.63	0.24	0.009
Sexual	76	42.7 (34.4)	49.8 (46.5)	0.76	−0.18	0.49
Violent	172	45.7 (39.1)	38.3 (30.8)	−1.40	0.22	0.16

*Note: t‐*test for independent samples, significance two‐sided.

There was a positive treatment effect on general reoffending, as the recidivists in the TG reoffended significantly later. For violent reoffending, we found a similar tendency. For sexual reoffending, there was a nonsignificant tendency in the opposite direction; that is, untreated individuals relapsed a little later than those in the TG. These findings were confirmed in more detailed Cox regression analyses (figures not shown here for reasons of space). After control of the Static‐99 risk level, there was a significant treatment effect (Exp(*β*) = 0.76, *p* < 0.01) for general reoffending and a similar tendency for violent reoffending (Exp(*β*) = 0.83, *p =* 0.22). The nonsignificant effect for sexual reoffending went in the opposite direction (Exp(*β*) = 1.55, *p* = 0.08), that is, the survival curves for the CG run slightly above the TG.

### 3.2 Differences Between Types of Offenders (Research Question 4)

Of the 396 individuals who had been sentenced for child abuse, 64.3% were treated in prison, whereas treatment for the group of 235 individuals with adult victims (i.e., rape offences) was less frequent (47.7%). Offenders with child victims were mainly treated in STUs (65.2%). A quarter received individual therapy and 9.8% received group therapy in regular prisons. The respective percentages for persons with adult victims were 56.6%, 23.0% and 20.4%. Table 3 shows the results on treatment effects for the two main types of offenders.^2^

**TABLE 3:** Recidivism rates (%) for the two main types of index offences.

	*n*	General	Sexual	Violent	Very severe
Child victims					
Treated	396/371	29.4/28.0%	8.1/7.5%	6.1/6.0%	5.1/5.2%
Untreated	220/219	30.0/27.8%	5.5/6.5%	6.8/6.9%	5.5/6.0%
Adult victims					
Treated	235/228	44.3/45.2%	6.0/5.0%	24.3/20.5%	9.4/8.6%
Untreated	258/254	50.0/49.2%	3.1/7.6%	23.3/24.7%	9.7/12.2%

*Note:* Figures to the left of the slash are without matching; on the right, PSM is balanced; all comparisons between TG and CG are nonsignificant (*p* > 0.05).

Individuals with adult rape victims had higher reoffending rates in various outcome criteria. The respective comparisons between TGs and CGs revealed no significant differences.

### 3.3 Differences Between the STUs (Research Questions 5 and 6)

As the institutional context is relevant for rehabilitation (Auty and Liebling 2020; Carl et al. 2019; Lösel 2018), we gathered differential data on the seven STUs. Table 4 shows the recidivism rates of the various institutions and the Static‐99 risk level of their inmates. We also present data on the dropout rates in the respective institutions. Because of our intent‐to‐treat analyses, the individuals who did not complete treatment were included in the TGs (for a more detailed analysis on treatment dropout and recidivism, see Carl and Lösel 2021).

**TABLE 4:** Recidivism rates, dropout rates and risk scores in the various STUs.

STU	General (%)	Sexual (%)	Violent (%)	Very severe (%)	Dropout^a^	Static‐99 *M* (SD)
A	61.9	16.7	28.6	16.7	39.0	4.10 (2.32)
B	23.6	5.6	6.9	4.2	12.7	1.37 (1.34)
C	51.9	9.3	18.5	13.0	18.9	3.07 (2.47)
D	18.3	7.5	7.5	6.5	15.2	1.61 (1.57)
E	53.2	11.3	29.0	12.9	4.9	2.73 (1.67)
F	20.0	4.0	4.0	4.0	40.0	2.14 (2.61)
G	35.5	5.3	6.6	2.6	21.3	2.32 (1.99)

*Note:* The sample sizes in the various STUs varied between 25 and 93 (*M* = 60.6). The detailed figures are not reported here because we had guaranteed anonymity to the institutions, and it would be possible to infer them from the respective figures. We informed each STU about its own results.

^a^ Individuals who did not complete treatment were counted as treated.

The reoffending rates varied substantially between the STUs. They were significantly different for general recidivism (χ^2^(6) = 46.3, *p* < 0.001), violent recidivism (χ^2^(6) = 32.31, *p* < 0.001) and very severe recidivism (χ^2^(6) = 13.35, *p* < 0.05), but not for sexual recidivism (χ^2^(6) = 7.00, *p* = 0.32). In additional analyses we used a meta‐analytic integration and an inverse‐variance weighting of the STU subsamples. There was heterogeneity in general reoffending (*Q*(6) = 52.58, *p* < 0.001), violent reoffending (*Q*(6) = 26.15, *p* < 0.001) and very severe reoffending (*Q*(6) 12.62, *p* < 0.05), but not for sexual reoffending (*Q*(6) = 5.55, *p* = 48). The STUs differed in the dropout rates (*Q*(6) = 52.58, *p* < 0.001) and in the scores in the Static‐99 risk instrument (Q(6) = 60.99, *p* < 0.001). When we used the Static‐99 as a control variable, the heterogeneity for violent recidivism remained significant (*p* = 0.02), which was mainly due to different proportions of individuals who had committed rape as the index offence.

We also compared the different STUs in terms of their institutional climate. In the Essen Climate Evaluation Schema (EssenCES), there was mainly similarity, but also some discrepancy between the ratings by the inmates and staff. For example, in the scale on *Experienced Safety*, there were significant mean differences in STU F (Mann–Whitney U test, *U* = 45.0, *z* = 2.72, *p* < 0.01) and STU G (*U* = 14.0, *z* = 3.54, *p* < 0.001), with higher scores in the prisoners' ratings. The scale *Inmates' Mutual Cohesion and Suppor*t showed significant mean differences in STU A (*U* = 89.5, *z* = −2.97, *p* < 0.01, staff > inmates) and STU E (*U* = 41.0, *z* = 2.18, *p* < 0.05; inmates > staff). *Support from Staff* was mostly rated lower by the inmates than by the staff members. In addition to the similarities and discrepancies within the specific STUs, we also analysed the variation of mean scores between the STUs. For the staff ratings in the EssenCES scales, there were no significant differences, which indicates rather homogeneous evaluations across the various institutions. In contrast, we found significant differences in the prisoners' evaluations of *Inmates' Mutual Cohesion and* *Support* (χ^2^(6) = 28.92, *p* < 0.001, lowest in A), *Experienced Safety* (χ^2^(6) = 50.59, *p* < 0.001, highest in G and F), *Support from Staff* (χ^2^(6) = 23.29, *p* < 0.001, highest in A) and the *CES Total Score* (χ^2^(6) = 27.12, *p* < 0.001, highest in G).

Most relations between the scores in the institutional climate scales and other variables were small and partially inconsistent. However, some findings were consistent and practically relevant: Older inmates had higher scores for *Inmates' Mutual Cohesion and* *Support, Experienced Security* and the *Total Score* than younger ones (*r =* 0.18, 0.21 and 0.22; all *p* < 0.05). Inmates of an STU that accommodated not only individuals who had committed sexual offences but also groups with nonsexual violence had lower scores in *Inmates' Mutual Cohesion and* *Support* (*r* = −0.35, *p* < 0.001), *Experienced Security* (*r* = −0.45, *p* < 0.001) and the *CES Total Score* (*r* = −0.30, *p* < 0.01) than individuals who were placed in the STUs that specialised in sexual offending. The relation with *Support from Staff* was *r* = 0.08 (nonsignificant).

The inmates' ratings of the institutional climate correlated with their evaluation of treatment effectiveness. The respective coefficients were *r* = 0.32 for *Inmates' Mutual Cohesion and* *Support*, 0.35 for *Experienced Safety*, 0.62 for *Support from Staff* and *0*.*57* for the *CES Total Scale* (all *p* < 0.001). There were also various relations between the climate evaluations in the various STUs and objective data. After control of the risk level in the respective STUs, the prisoners' ratings of *Support from Staff* correlated with lower rates of violent recidivism (*z* = −0.2.85, *p* < 0.01). Similarly, more positive staff evaluations of the *Inmates' Mutual Cohesion and* *Support* were related to lower violent recidivism in the respective STUs (*z* = −0.2.20, *p* < 0.05). There was also a lower general reoffending rate for higher scores in the staff ratings of *Support from Staff* (*z* = −1.95, *p* = 0.05) and a tendency of lower violent recidivism for higher staff ratings in the scale *Support from Staff* (*z* = −1.79, *p* = 0.07). The staff evaluations in the *EssenCES Total Score* and in the scale *Support from Staff* also correlated with the dropout rates; *z* = 2.22 (*p* < 0.05) and *z* = 3.10 (*p* < 0.01). Contrary to what could be expected, a more positive climate was associated with higher dropout rates.

### 3.4 Relapse Prevention by Treatment After Release (Research Question 7)

For a comprehensive treatment, it is important to prepare for resettlement and relapse prevention after release from prison. As described in the introduction, STUs typically care for this more intensively than regular prisons. In addition, for inmates with severe sentences, Bavaria has established specialised institutions in various communities that deliver treatment for offenders after their custodial sentence. This ambulatory treatment usually involves individual sessions with psychotherapists on a weekly or biweekly basis, accompanied by social work. In our total sample, 77% got a legal order for ambulatory treatment. A hierarchical logistic regression analysis showed that this order was more frequent for individuals who were younger, had only child victims and were already treated in custody. After controlling other covariates, a treatment order was related to less general reoffending among the untreated individuals (odds ratio OR = 0.51, *p* < 0.05). In the group with some individual or group treatment in regular prisons, there was a tendency of less general recidivism (OR = 0.52, *p* < 0.10). Results for violent recidivism were similar for untreated individuals (OR = 0.35, *p* < 0.05) and those with treatment in regular prisons (OR = 0.28, *p* < 0.05). Surprisingly, there was no effect of the relapse prevention treatment order for persons who were released from STUs. For sexual recidivism, the figures were too small for meaningful analyses.

## 4 Discussion

The main aim of this study was a well‐controlled evaluation of the effectiveness of the complex routine treatment of individuals who had committed sexual offences in social‐therapeutic prisons. This kind of intervention is partially similar to structured hierarchical therapeutic communities that showed positive effects for offenders with drug abuse problems (Lipton 1998). Beyond continental Europe, these approaches are rarely applied to individuals who committed sexual offences but seem to be promising for this group (Ware et al. 2010). This approach follows recommendations for broader interventions beyond isolated SOT programmes because the latter showed no or very small effects on sexual recidivism when delivered in prisons (Mews et al. 2017; Olver et al. 2012; Schmucker and Lösel 2017). In addition to the basic aim, we wanted to evaluate the influence of more specific factors and conditions, in particular different outcome variables, types of offenders, variations between included prisons, institutional climate and treatment after release for relapse prevention. Our research addressed both basic (causal?) questions of effectiveness as well as more descriptive issues with relevance for practice. The latter follows the concept that evaluation is not a one‐time assessment but a process by which a system can learn about itself (Cronbach et al. 1980).

Our PSM design formed balanced TGs and CGs that came close to an RCT, which for legal and practical reasons is rarely possible in the evaluation of custodial treatment for individuals who had committed serious sexual offences. The long‐lasting, complex treatment in STUs showed minimally but nonsignificantly lower rates of sexual reoffending in the TG. This may have been due to two main issues: In accordance with recent international trends, the sexual reoffending rates were generally low (ca. 4%–8%). That led to a ‘floor effect’ and reduced the possibility of detecting statistically significant differences between TGs and CGs. A second explanation refers to the composition of our TGs, where individuals with child victims were most frequent. Although the documentation of the Criminological Service of the Bavarian correctional system did not contain fully complete diagnoses, the presence of individuals with a paraphiliac or sexual deviance disorder may be a second reason for the lack of effects. This interpretation is supported by our analyses of the times of first reoffending after release. Sexual reoffending occurred somewhat earlier in the TG than in the CG (Table 2) and the Cox regression ‘survival curves’ were slightly below the CG. However, we should not speculate too much because all differences were nonsignificant. The same applies to the effects of treatment in regular prisons, where we found similar results on sexual reoffending to those for the STUs. Nonsignificant treatment effects on sexual reoffending are in agreement with other research on custodial interventions (e.g., Grady et al. 2017). However, there are also some more positive findings in the literature (e.g., Olver et al. 2020), and our study contained at least some promising messages: There was a stronger reduction of harm in the TG's sexual reoffending and a tendency of lower sexual reoffence rates in the more recent cohorts. If the latter represents a longer trend, it may indicate improved experience in specific treatments for sexual offenders (but societal changes or other factors after release may also play a role).

In contrast to sexual reoffending, there was a significant overall treatment effect on general reoffending and also in the recent cohort. This is in agreement with the literature. A similar tendency existed for treatment in regular prisons. Some of the above‐mentioned reasons may be relevant for these findings. Again, we refrain from speculating too much about reasons for nonsignificant findings and only underline the lower reoffending rates in the more recent cohort. A similar pattern resulted for the time at first reoffence. This was significantly shorter for general reoffending of untreated individuals, but for sexual reoffending, we observed a small nonsignificant opposite tendency.

The recidivism rates for all offences, violent offences and very severe offences were much lower for individuals who had abused children than for those with adult victims, that is, rape index offences (Table 3). For sexual reoffending, an opposite trend existed. In the group with child victims, the rates of sexual, violent and very severe recidivism were rather low (< 10%). This was also the case for sexual and very severe reoffending in the group of rapists. The comparison of PSM‐balanced TGs and CGs revealed no significant treatment effect. Although the low base rates may be an explanation here, there was also no significant treatment effect for outcomes with higher base rates. We assume that our control of risk and many other confounded variables reduced artificial influences and led to a true estimation of (non)effects. This is in accordance with our research on persons with ‘mixed’ sexual offences (Link and Lösel 2021) who were similar to those who had attacked only adult victims.

It is a strength of this study that we got data for different STUs. As Table 4 shows, there were substantial differences in recidivism between them. In all outcome categories, the STU with the highest reoffending rates was several times above the STU with the lowest rates. If our study had addressed only one STU, the results would have been misleading. This underlines the request for multicentre trials in psychiatry and other fields of medicine. The large differences between STUs could mainly be ‘explained’ by differences in the Static‐99 risk level of the inmates. Only for violent recidivism did a significant difference remain after controlling for risk. As in other countries, there are different categories of prisons (e.g., Categories A, B, C and D in Great Britain), and the Bavarian STUs are partially related to these differentiations.

Beyond the risk level, we also found that the local institutional climate was of some relevance. Our assessment of various dimensions of the social climate in the institutions gathered views from both inmates and staff members. The response rates were good so that we can assume a reliable picture of the assessed climate features. However, although organisational data and staff interviews showed much stability over time, we cannot be sure whether previous cohorts would have had the same experiences and evaluations. With this limitation in mind, the climate data seemed to be relevant for practice and were also appreciated when we presented them in workshops to practitioners. The mean scores were in a middle range so that most inmates did not use the survey for sweeping complaints. The evaluations from the inmates and staff members were rather similar, although the latter had a more favourable view on some dimensions (e.g., on support from staff and inmates' mutual cohesion and support). There were also more significant differences between the STUs in the inmates' scores than in the staff scores.

Even after statistically controlling for risk, we found various significant relations between reoffending and climate features. For example, both inmates' and staff’s ratings of support from the staff correlated with lower reoffending rates for general or violent recidivism. Although we only assessed seven different STUs, these data support broader findings on relations between the social climate in prisons and recidivism (e.g., Auty and Liebling 2020). It would be interesting to relate individual climate experiences with reoffending, but for reasons of data privacy, the respective analyses can only be carried out on the institutional level (Auty and Liebling 2024). In our study, we also found that climate features were related to the dropout rates in different institutions. Perhaps surprisingly, some STUs with higher dropout rates after rather long treatment durations showed more positive total scores in the EssenCES. This may have been due to an institutional policy that suspended some misbehaving or no longer motivated individuals to retain favourable therapeutic conditions. Similar strategies existed in international practice when individuals with high scores on the Psychopathy Checklist‐Revised (PCL‐R) were discriminated against by exclusion from any kind of treatment (see Lösel 1998). However, there are now more promising approaches (e.g., Polaschek 2014).

As our project aimed for a relatively comprehensive evaluation of treatment practice, we also addressed community treatment centres after release. Our analysis showed that individuals who had victimised children and had been treated in custody were a preferred target group for this kind of relapse prevention. This focus was in accordance with the particular sensitivity for child abuse in the recruitment for treatment in STUs. Individuals who were not treated in custody showed lower rates of general and violent reoffending than those without an order, but there was no effect for inmates who had received treatment in STUs. Unfortunately, we only had basic data on the treatment order and not on details of the ambulatory treatment. According to a current project of Martin Schmucker at our institute, the ambulatory treatment approach is based on RNR. However, as the aftercare is concentrated in a few cities, there are practical problems of clients' travelling and communication with the therapeutic staff in the STUs. Beyond these details, our results suggest that a focus on former STU inmates may not be an optimal strategy. Perhaps individuals who had already received intensive custodial programmes may feel some ‘treatment satiation’. In addition, new therapeutic alliances need to be developed so that a booster effect after custodial treatment should not simply be assumed.

Overall, custodial treatment seems to have partially desirable effects, but more on general than on sexual reoffending. The low sexual reoffending rates in both treated and untreated groups fit the long‐term decrease of sexual recidivism in many countries. This is a welcome development, although it makes proof of desirable treatment effects difficult. Our results on the low reoffending rates and reduced harm of reoffences are also in accordance with longitudinal research that shows much less continuity in most sexual offending than is assumed in the public and mass media (Jennings 2015; Lussier et al. 2021). Although sexual offending is of particular interest in the general population, our findings endorse the cautious conclusion that treatment in criminal justice systems should not focus too much on sexual offending but allocate sufficient treatment resources for other seriously violent individuals who also cause much harm to victims.

Beyond treatment studies, it is also necessary to investigate the complex influences on recidivism at the societal level. For example, the increased sensitivity to sexual offences and specific early prevention programmes may play a role in individual risk considerations. Whether easy access to pornography on the internet may have a cathartic or triggering influence is not yet clear (Holt et al. 2021). The same applies to the partially promising control of prostitution in some contexts. These and other influences on the macro level require more research at the individual level.

### 4.1 Limitations

Although our study has various strengths, it also has limits. *First*, our outcome data were restricted to official recidivism. Other data such as self‐reports may have led to partially different findings. However, self‐reports are mostly related to official crimes, although not in all groups (Joliffe et al. 2003). Self‐reports are also less suitable and valid for serious sexual offending with frequent denial (Lösel and Dillig 1973). *Second*, although there are data on stability, our assessment of institutional climate may not be generalisable to earlier cohorts. *Third*, we could not directly assess characteristics of offenders who had been incarcerated long ago. We gratefully used the rather comprehensive and overall thorough reports from staff members in the regular data entry form of the Bavarian Criminological Service. These were not 100% complete and, according to feedback from the Service, perhaps sometimes not fully valid. This was particularly the case for untreated persons and those who received some treatment in regular prisons. *Fourth*, as an RCT was not possible, we applied PSM techniques to reach equivalence between the TGs and CGs. Although PSM is widely used in treatment evaluations, there are also critical aspects of these methods (e.g., Jann 2017; King and Nielsen 2019). We tried to reduce some bias by using a substantial number of variables that are theoretically and empirically relevant in criminology and our field of research (Luellen et al. 2005). We also compared PSM with another matching procedure and found more consistent results with PSM (Lösel et al. 2020). *Fifth*, we could not directly assess details of the *development* of the individuals in our study before, during and after incarceration. This is similar in most evaluations in our field and does not allow conclusions about change *processes*. *Sixth,* partially related to the latter point, we could not disentangle specific influences of parts of the complex social‐therapeutic treatment package that had various elements. This is again a general problem that is particularly relevant for complex therapeutic communities and when programmes fail. Future micro‐evaluations of specific modules and qualitative studies can shed light on this.

## Funding

This work was supported by Bayerisches Staatsministerium der Justiz.

## Data Availability

The data that support the findings of this study are available from the corresponding author upon reasonable request.

## Endnotes

^1^
See also the portfolio of accredited offending behaviour programmes in England and Wales: https://www.gov.uk/guidance/offending‐behaviour‐programmes‐and‐interventions.

^2^
Exhibitionism as a sole category was negligible, as our samples consisted of individuals with severe prison sentences. We also investigated a group of individuals with ‘mixed’ types of reoffending, that is, both child and adult victims. In another study, we compared these individuals with groups who perpetrated only child or only adult victims (see Link and Lösel 2021). Offenders with both types of victims are sometimes labelled as potential ‘psychopaths’. However, we showed that the ‘mixed offenders’ were rather similar to individuals who had only adult victims, which is in line with a recent meta‐analysis (Williams et al. 2026).

## References

[cbm70029-bib-0001] Abracen, J. , J. Looman , M. Ferguson , L. Harkins , and D. Mailloux . 2011. “Recidivism Among Treated Sexual Offenders and Comparison Subjects: Recent Outcome Data From the Regional Treatment Centre (Ontario) High‐Intensity Sex Offender Treatment Programme.” Journal of Sexual Aggression 17, no. 2: 142–152. 10.1080/13552600903511980.

[cbm70029-bib-0002] Andrews, D. A. , J. Bonta , and S. Wormith . 2011. “The Risk‐Need‐Responsivity (RNR) Model: Does Adding the Good Lives Model Contribute to Effective Crime Prevention?” Criminal Justice and Behavior 38, no. 7: 735–755. 10.1177/0093854811406356.

[cbm70029-bib-0003] Austin, P. C. 2011. “An Introduction to Propensity Score Methods for Reducing the Effects of Confounding in Observational Studies.” Multivariate Behavioral Research 46, no. 3: 399–424. 10.1080/00273171.2011.568786. 21818162 PMC3144483

[cbm70029-bib-0004] Austin, P. C. , and E. A. Stuart . 2015. “Moving Towards Best Practice When Using Inverse Probability of Treatment Weighting (IPTW) Using the Propensity Score to Estimate Causal Treatment Effects in Observational Studies.” Statistics in Medicine 34, no. 28: 3661–3679. 10.1002/sim.6607. 26238958 PMC4626409

[cbm70029-bib-0005] Auty, K. M. , and A. Liebling . 2020. “Exploring the Relationship Between Prison Social Climate and Reoffending.” Justice Quarterly 37, no. 2: 358–381. 10.1080/07418825.2018.1538421.

[cbm70029-bib-0006] Auty, K. M. , and A. Liebling . 2024. “What Is a ‘Good Enough’ Prison? An Empirical Analysis of Key Thresholds Using Prison Moral Quality Data.” European Journal of Criminology 21, no. 5: 725–753. 10.1177/14773708241227693.

[cbm70029-bib-0007] Carl, L. , L. Lauchs , M. Schmucker , and F. Lösel . 2019. “‘Benchmarking’ in der Sozialtherapie: Vergleiche zwischen sozialtherapeutischen Abteilungen für Sexualstraftäter in Bayern [Benchmarking in Social Therapy: Comparisons Between Sociotherapeutic Institutions for Sex Offenders in Bavaria].” Forensische Psychiatrie, Psychologie, Kriminologie 13, no. 4: 371–379. 10.1007/s11757-019-00553-4.

[cbm70029-bib-0008] Carl, L. , and F. Lösel . 2021. “Dropout in Sexual Offender Treatment: Relations to Criminal History, Offence Characteristics, Treatment Duration, and Recidivism.” Criminal Behaviour and Mental Health 31: 1–15. 10.1002/cbm.2220. 34689361

[cbm70029-bib-0009] Cronbach, L. J. , S. R. Ambron , S. M. Dornbusch , et al. 1980. Toward a Reform of Program Evaluation. Jossey Bass.

[cbm70029-bib-0010] Cullen, E. , and L. J. R. Woodward . 1997. Therapeutic Communities for Offenders. Wiley.

[cbm70029-bib-0011] Farrall, S. , and A. Calverley . 2005. Understanding Desistance From Crime. Open University Press.

[cbm70029-bib-0067] Fazel, S. , C. Hurton , M. Burghart , M. DeLisi , and R. Yu . 2024. “An Updated Evidence Synthesis on the Risk‐Need‐Responsivity (RNR) Model: Umbrella Review and Commentary.” Journal of Criminal Justice 92. https://doi.101016/j.crimjus.2024.102197.

[cbm70029-bib-0013] Gannon, T. A. , M. E. Olver , J. S. Mallion , and M. James . 2019. “Does Specialized Psychological Treatment for Offending Reduce Recidivism? A Meta‐Analysis Examining Staff and Program Variables as Predictors of Treatment Effectiveness.” Clinical Psychology Review 73: 101752. 10.1016/j.cpr.2019.101752. 31476514

[cbm70029-bib-0014] Genders, E. , and E. Player . 1995. Grendon: A Study of a Therapeutic Prison. Clarendon Press.

[cbm70029-bib-0015] Genders, E. , and E. Player . 2010. “Therapy in Prison: Revisiting Grendon 20 Years on.” Howard Journal of Criminal Justice 49, no. 5: 431–450. 10.1111/j.1468-2311.2010.00635.x.

[cbm70029-bib-0016] Grady, M. D. , D. Edwards , and C. Pettus‐Davis . 2017. “A Longitudinal Outcome Evaluation of a Prison‐based Sex Offender Treatment Program.” Sexual Abuse 29, no. 3: 239–266. 10.1177/1079063215585731. 25964025

[cbm70029-bib-0017] Hanson, R. K. , and D. Thornton . 2000. “Improving Risk Assessments for Sex Offenders: A Comparison of Three Actuarial Scales.” Law and Human Behavior 24, no. 1: 119–136. 10.1023/a:1005482921333. 10693322

[cbm70029-bib-0018] Holper, L. , A. Mokros , and E. Habermeyer . 2024. “Moderators of Sexual Recidivism as Indicator of Treatment Effectiveness in Persons With Sexual Offense Histories: An Updated Meta‐Analysis.” Sexual Abuse 36, no. 3: 255–291. 10.1177/10790632231159071. 36927218 PMC10880427

[cbm70029-bib-0019] Holt, K. , J. Kissinger , C. Spickler , and V. Roush . 2021. “Pornography Use and Sexual Offending: An Examination of Perceptions of Role and Risk.” International Journal of Offender Therapy and Comparative Criminology 68, no. 6–7: 613–637. 10.1177/0306624X211049183. 34634958

[cbm70029-bib-0020] Jann, B. 2017. Why Propensity Scores Should Be Used for Matching. German Stata Users' Group Meetings 2017. Stata Users Group.

[cbm70029-bib-0021] Jennings, W. G. 2015. Innovations and Advancements in Sex Offender Research. Routledge.

[cbm70029-bib-0022] Joliffe, D. , D. P. Farrington , J. D. Hawkins , R. F. Catalano , K. G. Hill , and R. Kosterman . 2003. “Predictive, Concurrent, Prospective and Retrospective Validity of Self‐Reported Delinquency.” Criminal Behaviour and Mental Health 13: 179–197. 10.1002/cbm.541. 14654870

[cbm70029-bib-0023] Kennard, D. 1998. An Introduction to Therapeutic Communities, 2nd ed. Routledge.

[cbm70029-bib-0024] King, G. , and R. Nielsen . 2019. “Why Propensity Scores Should Not Be Used for Matching.” Political Analysis 27, no. 4: 435–454. 10.1017/pan.2019.11.

[cbm70029-bib-0025] Kleinke, C. , M. Stemmler , J. Reinecke , and F. Lösel . 2011. “Efficient Ways to Impute Incomplete Panel Data.” Advances in Statistical Analysis 95, no. 4: 351–373. 10.1007/s10182-011-0179-9.

[cbm70029-bib-0026] Koehler, J. , and F. Lösel . 2025. “A Meta‐Evaluative Synthesis of the Effects of Custodial and Community‐Based Offender Rehabilitation.” European Journal of Criminology 22: 3–29. 10.1177/1477370824125650.

[cbm70029-bib-0027] Kuss, O. , M. Blettner , and J. Börgermann . 2016. “Propensity Score – Eine alternative Methode zur Analyse von Therapieeffekten [Propensity Score – An Alternative Method for Analyzing Treatment Effects].” Deutsches Ärzteblatt 113, no. 35–36: 597–603. 10.3238/arztebl.2016.0597. PMC5963493 27658473

[cbm70029-bib-0028] Lauchs, L. , E. Link , and F. Lösel . 2020. “Die Erfassung der Deliktschwere in Evaluationsstudien zur Straftäterbehandlung: Entwicklung und Anwendung eines strafrahmenorientierten Ansatzes [Measuring Offense Severity in Evaluation Studies on Offender Treatment: Development and Application of a Severity Index Based on Statutory Sentence Length].” Monatsschrift für Kriminologie und Strafrechtsreform 103, no. 4: 300–314. 10.1515/mks-2020-2060.

[cbm70029-bib-0029] Link, E. , and F. Lösel . 2021. “‘Mixed’ Sexual Offending Against Both Children and Adults: An Empirical Comparison With Individuals Who Exclusively Offended Against Child or Adult Victims.” Criminal Justice and Behavior 48, no. 11: 1616–1633. 10.1177/00938548211002882.

[cbm70029-bib-0030] Link, E. , and F. Lösel . 2022. “Development and Application of an Offense Severity Index in the Evaluation of Treatment of Individuals Convicted of Sexual Crimes.” Psychology, Crime & Law 29, no. 7: 722–739. 10.1080/1068316X.2022.2032054.

[cbm70029-bib-0031] Lipton, D. S. 1998. “Therapeutic Community Treatment Programming in Corrections.” Psychology, Crime & Law 4, no. 3: 213–263. 10.1080/10683169808520010.

[cbm70029-bib-0032] Lösel, F. 1998. “Treatment and Management of Psychopaths.” In Psychopathy: Theory, Research and Implications for Society, edited by D. J. Cooke , A. E. Forth , and R. B. Hare , 303–354. Kluwer Academic Publishers.

[cbm70029-bib-0033] Lösel, F. 2012. “Offender Treatment and Rehabilitation: What Works?” In The Oxford Handbook of Criminology, edited by M. Maguire and R. Morgan , 5th ed., 986–1016. Oxford University Press.

[cbm70029-bib-0034] Lösel, F. 2018. “Evidence Comes by Replication but Needs Differentiation: The Reproducibility Issue in Science and Its Relevance for Criminology.” Journal of Experimental Criminology 14, no. 3: 257–278. 10.1007/s11292-017-9297-z.

[cbm70029-bib-0035] Lösel, F. , and D. Bender . 2003. “Protective Factors and Resilience.” In Prevention of Adult Antisocial Behaviour, edited by D. P. Farrington and J. Coid , 130–204. Cambridge University Press.

[cbm70029-bib-0036] Lösel, F. , L. C. Carl , and E. Link . 2023. Evaluation der Behandlung von Sexualtätern im Strafvollzug: Eine umfassende multizentrische Studie [Evaluation of sexual offender treatment in prisons: A comprehensive multicenter study]. 1st ed. Nomos.

[cbm70029-bib-0037] Lösel, F. , and P. Dillig . 1973. “Zur Gültigkeit und Zuverlässigkeit einer schriftlichen Erhebung von Daten der Straffälligkeit jugendlicher Krimineller [On the Validity and Reliability of a Written Survey on Juvenile Delinquency Data].” Monatsschrift für Kriminologie und Strafrechtsreform 56, no. 4: 171–182. 10.1515/mks-1973-560403.

[cbm70029-bib-0038] Lösel, F. , and D. P. Farrington . 2012. “Direct Protective and Buffering Protective Factors in the Development of Youth Violence.” American Journal of Preventive Medicine 43, no. 2: 8–23. 10.1016/j.amepre.2012.04.029. 22789961

[cbm70029-bib-0039] Lösel, F. , P. Köferl , and F. Weber . 1987. Meta‐Evaluation der Sozialtherapie [Meta‐Evaluation of Socio‐Therapeutic Treatment]. Enke.

[cbm70029-bib-0040] Lösel, F. , E. Link , M. Schmucker , et al. 2020. “On the Effectiveness of Sexual Offender Treatment in Prison: A Comparison of Two Different Evaluation Designs.” Sexual Abuse: A Journal of Research and Treatment 32, no. 4: 452–475. 10.1177/1079063219871576. 31451086 PMC7218343

[cbm70029-bib-0041] Luellen, J. K. , W. R. Shadish , and M. H. Clark . 2005. “Propensity Scores: An Introduction and Experimental Test.” Evaluation Review 29, no. 6: 530–558. 10.1177/0193841x05275596. 16244051

[cbm70029-bib-0042] Lussier, P. , E. McCuish , and J. Cale . 2021. Understanding Sexual Offending: An Evidence‐Based Response to Myths and Misconceptions. Springer.

[cbm70029-bib-0043] Lussier, P. , E. McCuish , J. Proulx , S. Chouinard Thivierge , and J. Frechette . 2023. “The Sexual Recidivism Drop in Canada: A Meta‐Analysis of Sex Offender Recidivism Rates Over an 80‐Year Period.” Criminology & Public Policy 22, no. 1: 125–160. 10.1111/1745-9133.12611.

[cbm70029-bib-0044] Maguire, M. , D. Grubin , F. Lösel , and P. Raynor . 2010. “‘What Works’ and the Correctional Services Accreditation Panel: Taking Stock From an Inside Perspective.” Criminology and Criminal Justice 10, no. 1: 37–58. 10.1177/1748895809352651.

[cbm70029-bib-0045] Maruna, S. 2001. Making Good: How Ex‐Convicts Reform and Rebuild Their Lives. American Psychological Association.

[cbm70029-bib-0046] McGuire, J. , M. C. Seto , J. Davies , and F. Lösel . 2025. “Significantly Off Target: A Commentary on Fazel et al.’s (2024) Umbrella Review of Research on the Risk–Need–Responsivity Framework.” Journal of Experimental Criminology: online first. 10.1007/s11292-025-09707-3.

[cbm70029-bib-0047] Mews, A. , L. Di Bella , and M. Purver . 2017. “Impact Evaluation of the Prison‐Based Core Sex Offender Treatment Programme.” Ministry of Justice Analytical Series. https://www.gov.uk/government/publications/impact‐evaluation‐of‐the‐prison‐based‐core‐sex‐offender‐treatment‐programme.

[cbm70029-bib-0048] Netto, N. R. , J. M. Carter , and C. Bonell . 2014. “A Systematic Review of Interventions That Adopt the ‘Good Lives’ Approach to Offender Rehabilitation.” Journal of Offender Rehabilitation 53, no. 6: 403–432. 10.1080/10509674.2014.931746.

[cbm70029-bib-0049] Olver, M. E. , L. E. Marshall , W. L. Marshall , and T. P. Nicholaichuk . 2020. “A Long‐Term Outcome Assessment of the Effects on Subsequent Reoffense Rates of a Prison‐Based CBT/RNR Sex Offender Treatment Program With Strength‐Based Elements.” Sexual Abuse 32, no. 2: 127–153. 10.1177/1079063218807486. 30362904

[cbm70029-bib-0050] Olver, M. E. , T. P. Nicholaichuk , D. Gu , and S. C. P. Wong . 2012. “Sex Offender Treatment Outcome, Actuarial Risk, and the Aging Sex Offender in Canadian Corrections: A Long‐Term Follow‐Up.” Sexual Abuse: A Journal of Research and Treatment 25, no. 4: 396–422. 10.1177/1079063212464399. 23136142

[cbm70029-bib-0052] Olver, M. E. , S. C. P. Wong , and T. P. Nicholaichuk . 2009. “Outcome Evaluation of a High‐Intensity Inpatient Sex Offender Treatment Program.” Journal of Interpersonal Violence 24, no. 3: 522–536. 10.1177/0886260508317196. 18458350

[cbm70029-bib-0053] Polaschek, D. L. L. 2014. “Adult Criminals With Psychopathy: Common Beliefs About Treatability and Change Have Little Empirical Support.” Current Directions in Psychological Science 23, no. 4: 296–301. 10.1177/0963721414535211.

[cbm70029-bib-0054] Rettenberger, M. , T. Haubner‐MacLean , and R. Eher . 2013. “The Contribution of Age to the Static‐99 Risk Assessment in a Population‐Based Prison Sample of Sexual Offenders.” Criminal Justice and Behavior 40: 1413–1433. 10.1080/13218719.2020.1767714.

[cbm70029-bib-0055] Rosenbaum, P. R. , and D. B. Rubin . 1983. “The Central Role of the Propensity Score in Observational Studies for Causal Effects.” Biometrika 70, no. 1: 41–55. 10.1093/biomet/70.1.41.

[cbm70029-bib-0056] Schalast, N. , and M. Tonkin . 2016. The Essen Climate Evaluation Schema Essen CES. Hogrefe.

[cbm70029-bib-0057] Schmucker, M. , and F. Lösel . 2015. “The Effects of Sexual Offender Treatment on Recidivism: An International Meta‐Analysis of Sound Quality Evaluations.” Journal of Experimental Criminology 11, no. 4: 597–630. 10.1007/s112920159241-z.

[cbm70029-bib-0058] Schmucker, M. , and F. Lösel . 2017. “Sexual Offender Treatment for Reducing Recidivism Among Convicted Sex Offenders: A Systematic Review and Meta‐Analysis.” Campbell Systematic Reviews 13, no. 1: 1–75. 10.4073/csr.2017.8.

[cbm70029-bib-0059] Sherman, L. , P. W. Neyroud , and E. Neyroud . 2016. “The Cambridge Crime Harm Index: Measuring Total Harm From Crime Based on Sentencing Guidelines.” Policing: Journal of Policy Practice 10, no. 3: 171–183. 10.1093/police/paw003.

[cbm70029-bib-0060] Spöhr, M. 2009. Sozialtherapie von Sexualstraftätern im Justizvollzug: Praxis und Evaluation [Socio‐therapeutic treatment of sexual offenders in prisons: Routine practice and evaluation]. Forum Verlag.

[cbm70029-bib-0061] Ward, T. , and C. Stewart . 2003. “Criminogenic Needs and Human Needs: A Theoretical Model.” Psychology, Crime & Law 9, no. 2: 125–143. 10.1080/1068316031000116247.

[cbm70029-bib-0062] Ware, J. , A. Frost , and A. Hoy . 2010. “A Review of the Use of Therapeutic Communities With Sexual Offenders.” International Journal of Offender Therapy and Comparative Criminology 54, no. 5: 721–742. 10.1177/0306624x09343169. 19666834

[cbm70029-bib-0063] Williams, S. K. , D. El Chuck , and S. Stephens . 2026. “A Meta‐Analysis of Atypical Sexuality, Psychopathy, and Recidivism Associated With Victim Age Polymorphism.” Sexual Abuse 38. 10.1177/10790632261415817. PMC12916886 41579053

[cbm70029-bib-0064] Xu, S. , C. Ross , M. A. Raebel , S. Shetterly , C. Blanchette , and D. Smith . 2010. “Use of Stabilized Inverse Propensity Scores as Weights to Directly Estimate Relative Risk and Its Confidence Intervals.” Value in Health 13, no. 2: 273–277. 10.1111/j.1524-4733.2009.00671.x. 19912596 PMC4351790

[cbm70029-bib-0065] Zeccola, J. , S. F. Kelty , and D. Boer . 2021. “Does the Good Lives Model Work? A Systematic Review of the Recidivism Evidence.” Journal of Forensic Practice 23, no. 3: 285–300. 10.1108/JFP-03-2021-0010.

